# Hydroxytyrosol, a Component of Olive Oil for Breast Cancer Prevention in Women at High Risk of Cancer

**DOI:** 10.1155/ijbc/8831168

**Published:** 2025-01-21

**Authors:** Akshjot Puri, Zheng Yin, Sergio Granados-Principal, Joe Ensor, Liliana Guzman, Roberto Rosato, Hong Zhao, Stephen Wong, Lin Wang, Tejal Patel, Jenny C. Chang

**Affiliations:** ^1^Department of Hematology and Oncology, Houston Methodist Dr Mary and Ron Neal Cancer Center, Houston, Texas, USA; ^2^Department of Systems Medicine and Bioengineering, Houston Methodist Research Institute, Houston, Texas, USA; ^3^Department of Biochemistry and Molecular Biology 2, School of Pharmacy, University of Granada, Granada, Spain; ^4^GENYO, Centre for Genomics and Oncological Research, Pfizer/University of Granada/Andalusian Regional Government, Granada, Spain; ^5^Instituto de Investigacion Biosanitaria ibs.GRANADA, University Hospitals of Granada-University of Granada, Granada, Spain; ^6^Department of Biostatistics, Natera Inc, Austin, Texas, USA; ^7^Houston Methodist Research Institute, Weill Cornell Medicine, Houston, Texas, USA; ^8^Department of General Oncology, The University of Texas MD Anderson Cancer Center, Houston, Texas, USA

**Keywords:** breast density, DNA damage repair, hydroxytyrosol, olive oil, Wnt pathway

## Abstract

**Background:** This study evaluates the effects of hydroxytyrosol (HT), a component of olive oil, on mammographic breast density reduction. We explored effects of HT on Wnt *β*-catenin and other pathways involved in cancer stem cell renewal, DNA repair, cell proliferation, and differentiation.

**Methods:** Twenty-five milligrams per day oral dose of HT was given for 12 months in pre- and postmenopausal women at increased risk of breast cancer. Out of 51 patients enrolled, 41 completed the study. The annualized percent decrease in maximum mammographic volumetric breast density (max VBD%) between baseline (BL) and end of treatment (EOT) was analyzed. RNA sequencing (RNA-Seq) and multiplex analysis was performed on the breast biopsies to compare the BL with EOT samples.

**Results:** Max VBD% showed a nonsignificant change; however, in women 60 years or older, the max VBD% decrease was significant (3.7%, *p* = 0.0391), especially in those with high BL mammographic density. Using RNA-Seq, 3330 unique transcripts were identified (*p* < 0.05). Mitotic telophase/cytokinesis and DNA damage were upregulated, whereas Wnt, Notch, and oxidative stress–induced senescence pathways were downregulated (*p* < 0.05). These pathways were confirmed by NanoString nCounter where significant decrease in proliferative genes (RELA and CDK4) and Wnt pathway (R-HSA-195721 and R-HAS-201681) was observed (*p* < 0.05).

**Conclusions:** HT reduced breast density only in women over 60 years, especially in those with high BL breast density. HT also reduced proliferation and affected the Wnt signaling pathway. This study lays the foundation for future larger studies in exploring a natural compound with well tolerability and overall nontoxic profile for chemoprevention of breast cancer.

**Trial Registration:** ClinicalTrials.gov identifier: NCT02068092

## 1. Introduction

Breast cancer is the leading cause of cancer death among women aged 20–59 years. Prevention of breast cancer is feasible by using selective estrogen receptor (ER) modulators and aromatase inhibitors (AIs). However, their use is limited by their side effects and lack of effect on ER-negative breast cancers. The average lifetime risk of breast cancer in an American woman is one in eight, which is increased in women with a strong family history of breast cancer, germline BRCA 1 and 2 mutations, atypical hyperplasia or lobular carcinoma in situ (LCIS), and history of mantle irradiation for Hodgkin's disease [1]. Hence, it is imperative to identify more effective strategies that prevent all subtypes of breast cancer, lower toxicity, and target key pathways such as DNA damage repair and regulators of stem cell function such as the canonical Wnt pathway.

Several nutritional studies have demonstrated an inverse relationship between consumption of olive oil and incidence of breast cancer [2]. A meta-analysis evaluating relative risk (RR) estimates for high versus low olive oil consumption demonstrated reduction of the incidence of breast cancer by 38% (RR 0.62 95% confidence interval (CI) 0.44–0.88) [2]. These anticancer properties are thought to be mediated by phenolic compounds in olive oil, the most active component being hydroxytyrosol (HT). We have previously demonstrated that the potent antioxidant HT at a dose of 0.5 mg/kg for 5 days/week for 6 weeks by oral gavage decreased tumor volumes in rat models to the same degree as treatment with Adriamycin [3]. In addition, it demonstrated alteration of the Wnt/secreted frizzled-related protein (sFRP) signaling pathway and dose-dependent decrease of *β*-catenin and cyclin D1 protein levels. Similar findings were seen in epithelial-to-mesenchymal markers, a decrease in both Snail and Slug transcription factors, and an increase in protein levels of epithelial marker E-cadherin [3]. Various other antitumor studies with HT have reported significant antiproliferative and proapoptotic effects on different cancer cell lines. Also, clinical toxicity studies in healthy volunteers have proven that HT intake has no toxic effects [4].

Multicenter, randomized, crossover, controlled trials have studied the impact of olive oil at high, moderate, and low doses on the increase in high-density lipoprotein cholesterol levels. Based on this, 5 mg HT was approved by the European Food Safety Authority (EFSA) [5, 6]. A single oral dose of 2.5 mg/kg of HT is rapidly absorbed, with maximal plasma concentration detected in 13–16 min, and the levels were undetectable 2 h after administration [7] and no-observed-adverse-effect level of 500 mg/kg/day was concluded [8].

Studies have demonstrated a 4–6-fold increase in breast cancer risk for women with increased mammographic density for up to 8 years [9, 10]. Randomized studies have demonstrated that tamoxifen lowers mammographic breast density up to 4.3% yearly [11] and decreases the risk of breast cancer. It has been shown that with every 1% increase in breast density, there is a 2% increase in the RR of breast cancer [12]. This supports that mammographic density change can be used as a surrogate endpoint to study the effect of chemopreventive agents on breast cancer incidence. A previous epidemiological study in 2000 women showed about 30% reduction in breast density in women with high olive oil consumption (> 30.5 g/day) [13].

In this prospective study, the primary objective was to conduct a pilot breast cancer prevention study of HT administered for 1 year in both pre- and postmenopausal women at increased risk of breast cancer and evaluate reduction in mammographic density before and after drug therapy. The secondary objectives were to evaluate the effect of HT on the reduction in mammographic density in subgroups classified by age, menopausal status, and baseline (BL) breast density. The exploratory objectives of the study were to evaluate the ability of HT to modulate key regulatory pathways such as Wnt signaling, cancer stem cell (CSC), and apoptosis/proliferation pathways before and after drug therapy.

## 2. Methods

### 2.1. Subjects and Setting

**Figure 1:**
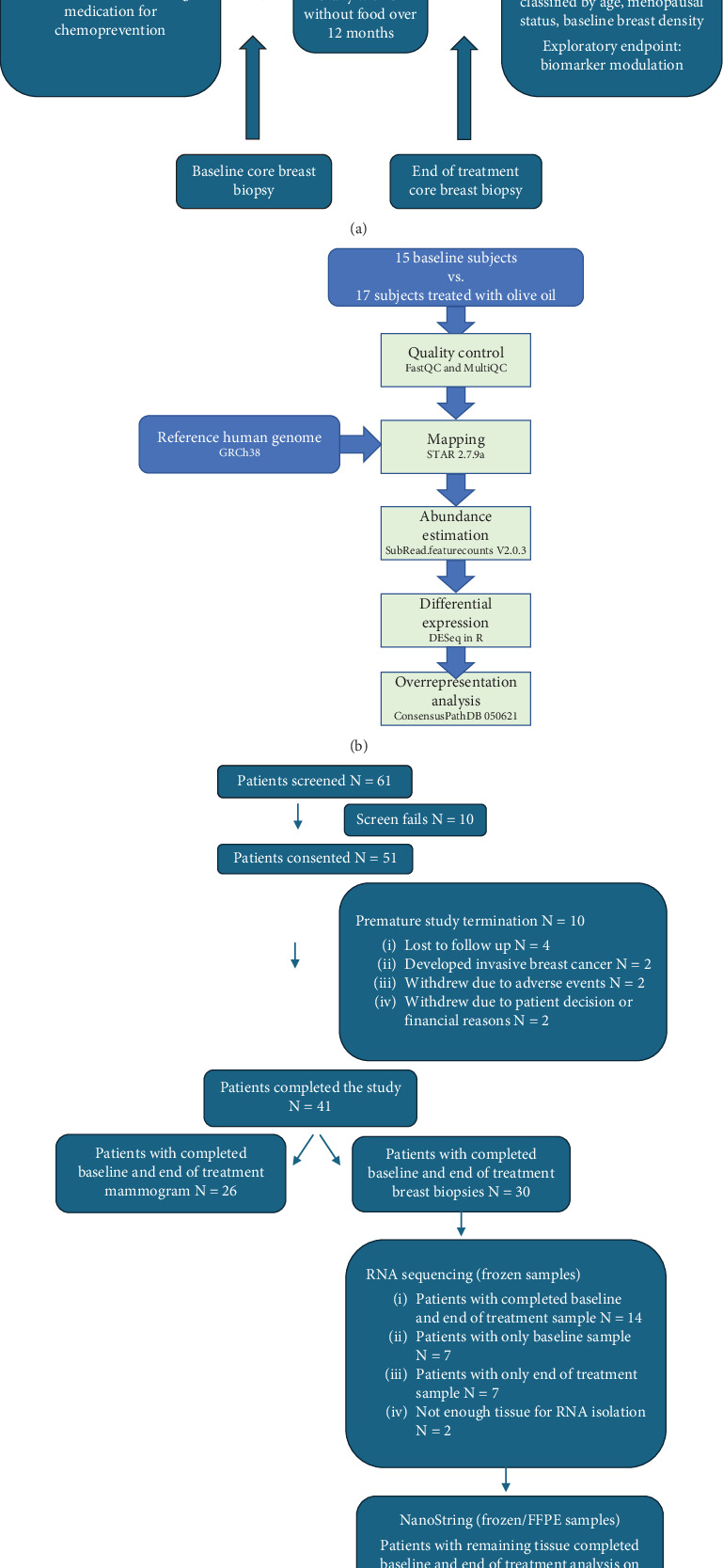
Schematic of this study. (a) Study design and (b) the workflow of RNA sequence analysis. (c) Consort diagram.

This study was a single-institution prospective study and included both pre- and postmenopausal women between ages 18 and 80 years at high risk of breast cancer who had declined standard-of-care antiestrogen medications and who received 25 mg/day oral HT capsules (PL Thomas & Co.) for a year. We included women with a modified 5-year Gail risk score of ≥ 1.7; known diagnosis of LCIS or resected ductal carcinoma in situ (DCIS); atypical ductal hyperplasia (ADH); atypical lobular hyperplasia (ALH); and germline mutations of BRCA 1, BRCA 2, PTEN (Cowden syndrome), and TP53 (Li-Fraumeni syndrome) genes; those with at least 10% probability of carrying BRCA mutation by BRCAPRO or similar model; and those with previous breast cancer (in complete remission for ≥ 5 years) with at least one nonirradiated breast. Women who were pregnant or breastfeeding and had a diagnosis of any active malignancy, bilateral breast implants (as they affect density measurements), and prior tamoxifen or raloxifene use within ≤ 28 days of enrollment or those with inability to take oral medication were excluded. The women underwent a mammogram before and after completion of the study drug. They were also offered an option of biopsy of normal breast tissue before starting and after completion of the study drug. All biopsies were directed to the upper outer quadrant of the breast, which is typically a dense region and were saved as formalin-fixed paraffin-embedded (FFPE) or frozen tissue for analysis.  Figure 1(a) shows the study design.

### 2.2. Measures and Outcomes

The mammographic density was assessed quantitatively after digitizing mammograms using FDA-approved Volpara software (VIS 3.2). Maximum volumetric breast density (max VBD%) which is calculated by fibroglandular breast volume divided by total breast volume (checked on the higher side, left or right) was used to obtain the study's endpoints. The primary endpoint was the annualized percent decrease in max VBD% between BL and end of treatment (EOT) with HT.

### 2.3. Follow-Up

The patients had a study visit at 4, 12, 24, and 52 weeks (30 days after the last dose of the study drug), which was counted from the start of treatment. There was a study visit at 56 weeks if the patients underwent the optional biopsy at the EOT. The study visit included a general history and physical exam, hematology and serum chemistry profiles, an assessment for safety, and concomitant medications and pill count. Patients with abnormal laboratory or clinical findings related to the study drug were followed until the condition resolved. CTC version 4.0 was used to grade toxicities. A drug holiday for maximum 12 consecutive weeks was allowed, and the drug was restarted at the same dose.

### 2.4. RNA Extraction

Total RNA was extracted from frozen biopsies according to the manufacturer's protocol using the RNeasy Lipid Tissue Mini Kit (Qiagen, #74804). Briefly, up to 100 mg of frozen tissue sections was homogenized in 1 mL QIAzol Lysis Reagent using the TissueLyser II (Qiagen). The homogenate was placed on the benchtop at room temperature (15°C–25°C) for 5 min to allow nucleoprotein complex dissociation. RNA was eluted with 40 *μ*L of RNase-free water and quantified using a Qubit 4 fluorometer (Invitrogen).

### 2.5. RNA Sequencing (RNA-Seq)

We generated RNA-Seq data from 15 BL and 17 EOT samples. After confirming the quality of the data using FastQC, we used STAR 2.7.9a to map the raw data to reference human genome version GRCH38 and generated BAM files. SubRead.featurecounts V2.0.3 was applied to the BAM files to estimate the abundance of genes within each sample.

R package DESeq was used to identify differentially expressed genes in EOT versus BL conditions and ConsensusPathDB v050621 to carry out overrepresentation analysis and identify canonical pathways enriched with EOT up- and downregulated genes, respectively (Figure 1(b)). There were two levels of statistical tests applied to the RNA-seq data, one at gene level and one at pathway level (Supporting Information (available here)).

### 2.6. NanoString nCounter Analysis

One hundred nanograms of RNA isolated from 16 paired BL and EOT samples was analyzed by NanoString nCounter Technology (Genomic and RNA Profiling Core, Baylor College of Medicine). Briefly, the collective CodeSet including target-specific reporters and capture probes was hybridized to regions of interest with covalently attached, target-specific sequences. Data import, quality control, and normalization of expression values were conducted with the nSolver version 4.0 (NanoString Technologies Inc.). Background subtraction from raw transcript counts was performed, normalized to housekeeper control genes, and log2 transformed before further analyses. Table S1 in the supporting information shows the 28 genes analyzed.

### 2.7. Statistical Analysis

This was a single-arm, pilot breast cancer prevention study of HT in women at increased risk of breast cancer. Our primary study endpoint was the annualized percent decrease in maximum VBD% between BL and EOT with HT. For each of the pre- and postmenopausal group of women, we assumed under the null hypothesis that 25% of women exhibit a response (at least a 25% reduction from BL) versus an alternative hypothesis equal to 45% based on data presented from Cuzick et al. [14]. Under these assumptions, a sample of 50 premenopausal and 50 postmenopausal women would have provided 85% power based on a two-sided test for a single proportion with 5% significance level for each group of women. However, we were unable to recruit the planned number of patients due to multiple barriers including lack of referral of eligible patients from peripheral and satellite hospitals, an underestimation of the number of patients who would refuse participation, and challenges in obtaining raw images and biopsies; hence, the trial had to be stopped early.

Demographics and pretreatment characteristics were collected and summarized for all patients using descriptive statistics. Mammographic density levels at BL and follow-up were summarized using means, standard deviations, and proportions. Change from BL levels of breast density was analyzed using paired tests while the proportion of women who exhibit at least a 10% absolute reduction from BL were calculated along with 95% binomial CIs for each group of women. Multivariate analysis to adjust for demographic/clinical characteristics, specifically age (< 60 or ≥ 60 years) or menopausal status (pre or post) and BL breast density (< 10% or ≥ 10%) was performed using logistic regression for analyzing proportion of women with response or using linear regression models for analysis of quantitative levels of mammographic density. Given the pilot nature of this study, these multivariate analyses are considered exploratory.

## 3. Results

Between December 2013 and September 2019, 61 women were screened for the trial and 51 were enrolled, out of which 41 patients completed the study. None of the 41 patients that completed the study had any treatment interruptions. In this intention-to-treat population (*N* = 51), 41 women were Caucasian, 4 were Hispanic, 4 were African American, and 2 were of Asian ethnicity. The study included women from ages 35 to 76 years (median age 54 years); other patient demographics are shown in Table 1. There were 26 patients who had both BL and EOT mammograms available for quantitative analysis; 30 patients provided BL and EOT breast biopsies for exploratory tissue analysis but only 28 patients had enough tissue for RNA extraction. Among these, 14 had both BL and EOT samples whereas 7 had only BL and 7 had only EOT samples. Only 15 BL and 17 EOT RNA samples met quality control parameters for further analysis. Sixteen patients had BL and EOT FFPE tissue which were used for NanoString analysis.  Figure 1(c) shows the consort diagram.

### 3.1. Mammographic Density Analysis

In all 26 patients, the mean decrease in max VBD% was relatively unchanged at −0.038% (95% CI −0.112–0.035, *p* value by signed rank test 0.49).

#### 3.1.1. BL Breast Density

For BL low versus high max VBD% of < 10% versus > 10%, 14 women had low breast density and 12 had high breast density. The difference in response to therapy between these two cohorts was not significant based on a pooled *t*-test (*p* = 0.4550) or a Wilcoxon rank-sum test (*p* = 0.6134) (Table S2).

#### 3.1.2. Menopausal Status

Out of the 26 patients, 10 were premenopausal and 16 were postmenopausal. The difference in response to therapy based on menopausal status was not significant based on a pooled *t*-test (*p* = 0.3183) or a Wilcoxon rank-sum test (*p* = 0.2180). A nonsignificant reduction in breast density in postmenopausal women with high BL max VBD% (> 10%) of −5.84% was observed (*p* = 0.318) (Table S3).

#### 3.1.3. Age

**Figure 2:**
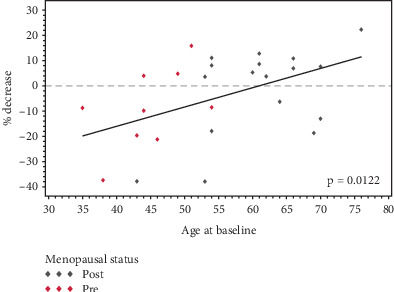
Percent decrease in breast density by age at baseline.

Age at BL is a significant predictor of the change in max VBD% (*p* = 0.0278); that is, the decrease in breast density was higher among older women. A simple linear regression was used to assess the bivariate relationship between percent decrease and age at BL. There was a significant positive linear trend (*p* = 0.0122) between age and percent decrease in breast density (Figure 2).

**Figure 3:**
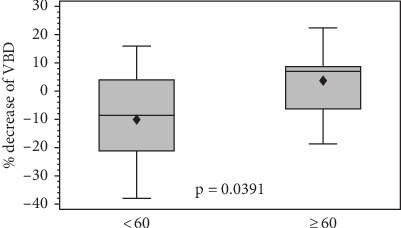
Box plot comparing percent decrease in breast density between age groups.

Among those aged 60 years or more and higher BL breast density (≥ 10% BL max VBD%), 80% responded with a decrease in density (Table S4). Multiple logistic regression model demonstrated that the odds of an older patient with higher breast density at BL to experience a decrease in breast density are 16 times greater than for a younger patient with less dense breasts at BL (*p* = 0.0795) (Table 2). As the variances are not significantly different between the two cohorts (*p* = 0.1817) based on a *F*-test of variance homogeneity, a two-sided pooled *t*-test was used to compare means between the two cohorts (*p* = 0.0391, Figure 3) and corroborated by a Wilcoxon rank-sum test (*p* = 0.0460).

### 3.2. RNA-Seq and NanoString nCounter Analysis

There were 3330 transcripts with *p* value ≤ 0.05 which were differentially expressed, 190 of these were upregulated, and 90 were downregulated in EOT versus BL by at least 1.5-fold (Supporting Information).

#### 3.2.1. Wnt Signaling Pathway Downregulated by HT

**Figure 4:**
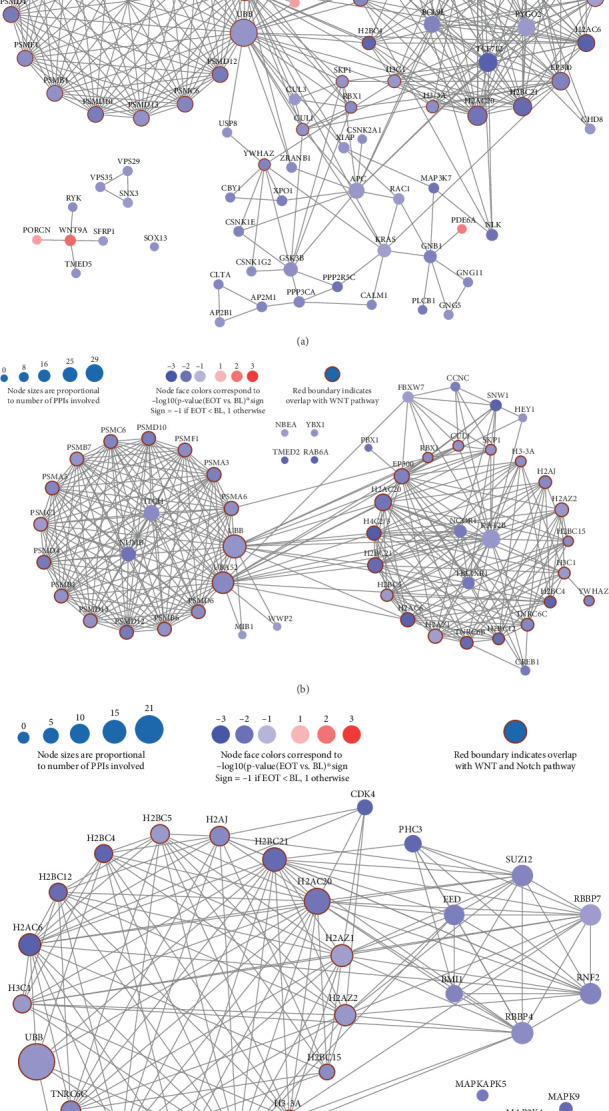
Reactome pathway. (a) “Signaling by WNT” is enriched with downregulated genes at the end of hydroxytyrosol. Eighty-two of 261 member genes R-HSA-195721 had *p* value ≤ 0.05 when comparing EOT to BL. Eighty one of the 82 genes were connected in 379 edges, with SOX13 being the only gene with no PPIs. 78 of the 82 genes are downregulated EOT. (b) “Signaling by Notch” is enriched with downregulated genes at the end of hydroxytyrosol. Fifty-four of 247 member genes R-HSA-157118 had *p* value ≤ 0.05 when comparing EOT to BL. Fifty of the 54 genes were connected in 323 edges. (c) “Oxidative stress–induced senescence” is enriched with downregulated genes at the end of hydroxytyrosol. Thirty-two of 126 member genes R-HSA-2559580 had *p* value ≤ 0.05 when comparing EOT versus BL. Thirty of the 32 genes were connected in 149 edges. All 32 genes were downregulated at EOT. Each node represents one gene, and each edge includes one high-confidence (confidence score > 0.7 from the STRING database). Node sizes are proportional to the number of high-confidence PPIs and the color of the node indicates the −log10 transformation for the *p* value between EOT and BL. Blue indicates downregulated at EOT while red means upregulation.

Two of the top 25 pathways overrepresented with EOT downregulated are related to Wnt signaling, that is, R-HSA-195721 (signaling by Wnt *p* value 0.0001587, *q*-value 0.001439) and R-HAS-201681 (TCF-dependent signaling in response to Wnt, *p* value 0.000151, *q*-value 0.001438) (Figure 4(a)) (Supporting Information).

#### 3.2.2. Notch Signaling Pathway Downregulated by HT

For R-HSA-157118, signaling by Notch was significantly overrepresented with EOT downregulated genes (*p* value 0.007983, *q*-value 0.02228), with 54 of 247 member genes that were downregulated (*p* value < 0.05) for EOT versus BL (Figure 4(b)) (Supporting Information).

#### 3.2.3. Oxidative Stress–Induced Senescence Pathway Downregulated by HT

For R-HSA-2559580, oxidate stress–induced senescence was significantly overrepresented with EOT downregulated genes (*p* value 0.0001669, *q*-value 0.001439), with 32 of 126 member genes that were downregulated (*p* value < 0.05) for EOT versus BL (Figure 4(c)) (Supporting Information).

#### 3.2.4. Cell Proliferation Signaling Significantly Downregulated After HT

**Figure 5:**
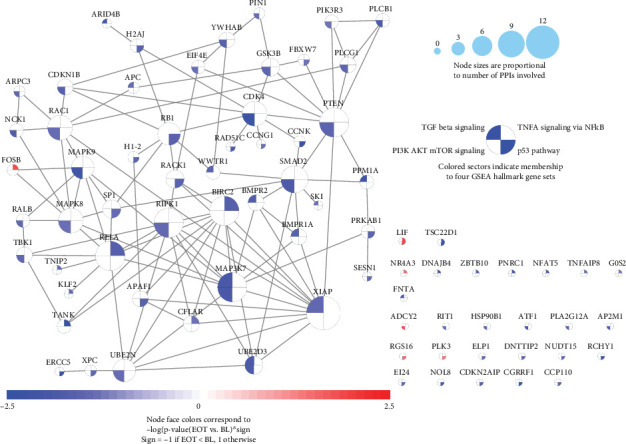
A subnetwork regulating cell proliferation is enriched with downregulated genes at the end of hydroxytyrosol. Seventy-eight member genes across four hallmark gene sets related to cell proliferations had *p* value ≤ 0.05 when comparing EOT versus BL. Each node represents one gene, and each edge includes one high-confidence (confidence score > 0.7 from the STRING database). Fifty-one of the 78 genes were connected in 110 edges. Node sizes are proportional to number of high-confidence PPIs. The face of each node was equally split into four sectors, with the sector in the first quadrant indicating the membership of the TNFA hallmark gene set, the second quadrant indicating the membership of the TGF_BETA gene set, the third quadrant indicating the membership of the PI3K_AKT_MTOR gene set, and the fourth quadrant for the P53 gene set. Each node was only colored in the sector representing the pathway containing the corresponding gene; the color of the node indicates the −log10 transformation for the *p* value between EOT and BL. Blue indicates downregulated at EOT while red means upregulation. All 32 genes are downregulated at EOT.

The four hallmark gene sets discussed in the Methods section were analyzed using DESeq and 78 unique genes with differential expression *p* value ≤ 0.05 (Figure 5), with 72/78 genes downregulated at EOT. There were 110 high confidence PPIs (confidence score > 0.7 from the STRING database) involving 51 of these 78 genes. Therefore, HT may impact cell proliferation by interacting with subnetworks across multiple pathways by downregulating the overall cell proliferation process.

### 3.3. Safety

Among the 10 patients that did not complete the study, two developed invasive breast cancer and two withdrew due to fatigue as a potential adverse event of HT. The other six were either lost to follow-up or made a personal decision to not continue the study. No remarkable changes in hematology or blood biochemistry were noted. The most common drug-related adverse events of any grade included abdominal pain (3.9%), bloating (3.9%), and headaches (3.9%). There were no drug-related deaths or other significant events (Table 3). One patient had a splenic infarct, and another had a brain aneurysm while on the drug while hospitalized for unrelated sickness. One patient had leukopenia, one had presyncope, and two patients had fatigue which were the only Grade 3 adverse events.

## 4. Discussion

Breast cancers with more aggressive clinical course tend to occur in younger patients, peripartum, African American ethnicity, and those with known genetic mutations such as BRCA 1 and TP53. There is a vast unmet need of chemopreventive agents with minimal side effects especially in women who refuse or are unable to tolerate antiestrogens due to side effects or those with ER^−^negative cancers. Our study did not meet its primary endpoint; that is, the breast density did not decrease in the intention-to-treat population after 12 months of treatment with HT. However, a subgroup analysis showed that women above 60 years of age and those with BL high max VBD% (≥ 10%) had more reduction in breast density as compared to those with low BL max VBD% (< 10%). Linear regression also showed increasing benefit and reduction in breast density with increasing age. Some reduction in breast density was seen in postmenopausal women as well, especially those with ≥ 10% max VBD but was not significant likely due to the small population size.

All patients that completed the study did not have any treatment breaks or serious side effects to HT. The two patients (3.9%) that withdrew from the study due to adverse events had Grade 3 fatigue. The most common adverse events noted were Grade 1–2 abdominal pain, bloating, and headaches. Almost 65% of the study population reported no adverse events whereas discontinuation rates in patients on tamoxifen and AIs have been reported to be 31%–73% [15]. Thus, this study demonstrates the overall safety and tolerability of HT.

Preclinical studies have demonstrated HT to have antiatherogenic, cardioprotective, anti-inflammatory, antiplatelet, and antimicrobial properties [4, 16–20]. This natural compound has been reported to regulate transcription factors (Nrf2, NFkB), cytokines (IL-6), prostaglandins, and COX-2 and respond to stress through the Wnt pathway [4, 16–20]. The WNT/*β*-catenin signaling pathway is an important regulator of the stem cell pathway controlling the development of the mammary gland during embryogenesis. This pathway is also frequently altered in invasive breast cancer and leads to a more aggressive phenotype and poor clinical outcome through the regulation of important cell events like proliferation, migration, differentiation, and apoptosis [21]. The sFRP family contains five secreted glycoproteins (sFRP1-5) that function as Wnt pathway inhibitor by directly interacting with both WNT ligands and FRP receptors. The sFRP family are silenced in several types of cancers including breast cancer [21]. The Wnt/*β*-catenin signaling pathway may also regulate stem cells [22]. It has been shown that sFRP4 generates reactive oxygen species (ROS) by blocking Wnt/*β*-catenin which results in oxidative stress–related damage. Our study demonstrates that a natural antioxidant compound can decrease the signaling in CSC pathways such as WNT and enhance DNA damage repair.

Studies have shown that Notch pathway activation is a major participant in breast cancer progression [23] and therapy resistance [24] and is the pathognomonic feature in triple-negative breast cancer (TNBC) [25]. Notch1 has a role in maintaining CSC stemness in TNBC, and inhibiting this signal not only has an inhibitory effect on this cancer subtype but also increases its chemosensitivity [26]. A major challenge in breast cancer treatment and prevention is to identify signaling pathways to target the tumor microenvironment. There is a need to study nontoxic alternatives to drugs such as PARP inhibitors. These can be essential as prophylaxis especially for patients at high risk of developing TNBC such as those with germline BRCA mutations by leveraging key signaling pathways like Notch. Similarly, obstructing oxidative stress in tumor microenvironment can lead to mitophagy and is a promising discovery for the development of future prophylactic strategies. Commonly used proliferation marker Ki67 has been successfully used in some trials as an endpoint to evaluate response to neoadjuvant endocrine therapy with a lower recurrent risk [27, 28]. Our study similarly showed an overall decrease in cell proliferation pathways after 1 year of HT treatment.

An important limitation of the study is the small sample size. Among the 41 patients that completed the study, only 28 patients had viable RNA from tissue biopsies and only 26 patients had both BL and EOT mammogram images available for analysis. Due to these small numbers, we were not able to correlate the changes in signaling pathways from RNA-Seq and NanoString with mammographic density changes for each patient. Another big limitation of the study is the lack of a concurrent control arm. Thus, it is difficult to draw a hard conclusion that the changes in breast density in older women were related to treatment with HT and not related to age, time, and other miscellaneous factors. Similarly, the biomarker changes from before and after HT could also possibly be due to changes over time or due to differences in tissue sampling. In addition, it is challenging to appreciate decrease in mammographic breast density in patients with BL low breast density. Our patient population consisted of 80% women of Caucasian origin and only 20% women belonging to other ethnicities. We were also unable to recruit the preplanned number of patients due to barriers including lack of referral of eligible patients from satellite hospitals; hence, the trial had to be stopped early. Counterintuitive to prior literature that suggested higher reduction in VBD with tamoxifen in premenopausal women versus AI in postmenopausal women with BL breast density ≥ 10% [29], our study shows a higher reduction in VBD in women ≥ 60 years after HT albeit the numbers are very small to make any specific conclusions in our study. This may be a function of the differences in the drug mechanisms of HT and AI or possibly differences in tissue sampling. Also, a large observational study in US women did not show a risk reduction in breast cancer in women who had higher versus those who had lower dietary intake of olive oil [30]. However, this study utilized regular household olive oil and not an extracted active component unlike our study.

## 5. Conclusion

Although this study did not meet its primary endpoint of significantly reducing mammographic breast density in the intention-to-treat population, breast density was reduced in women ≥ 60 years and with higher BL breast density without any increased toxicity. Studying the key pathways impacted by HT demonstrated downregulation of stem cell pathways such as Wnt and Notch and proliferative pathways. This pilot study lays foundation for future studies, with longer follow-up to analyze the benefit of HT in these high-risk women. It will be insightful to study the impact of factors such as age, ethnicity, obesity, and other comorbidities on breast microenvironments to understand why certain subgroups may benefit with HT.

## Figures and Tables

**Table 1:** Patient demographics (*N* = 51).

Age (years)	35–76 years (median age 54 years)
< 60	34
≥ 60	17
Ethnicity	(*N* = 51)
Caucasian	41
Hispanic	4
African American	4
Asian	2
Risk factors	(*N* = 51)
High Gail risk	25
Atypical hyperplasia (ADH or ALH)	22
Carcinoma in situ (LCIS or DCIS)	2
BRCA 2 mutation	2
Menopausal status	(*N* = 51)
Premenopausal	20
Postmenopausal	31

**Table 2:** Odds ratio from the multiple regression model.

**Factor**	**Reference level**	**Odds ratio**	**95% confidence limits**		**p** ** value**
Age ≥ 60 and density < 10%	Age < 60 and density < 10%	8.0	0.50	127.90	0.1415
Age < 60 and density ≥ 10%	Age < 60 and density < 10%	4.0	0.32	49.59	0.2805
Age ≥ 60 and density ≥ 10%	Age < 60 and density < 10%	16.0	0.72	354.78	0.0795

**Table 3:** Summary of adverse events.

**Event**	**All grades**	**G** **r** **a** **d** **e** ≥ 3	**Attribution to hydroxytyrosol**
	**Number of patients (percent)**	**Number of patients (percent)**	
Blood and lymphatic system disorders: leukopenia	1 (1.9%)	1 (1.9%)	Possible
Gastrointestinal disorders			
Abdominal pain	2 (3.9%)	0	Possible
Bloating	2 (3.9%)	0	Possible
Flatulence	1 (1.9%)	0	Possible
Splenic infarct	1 (1.9%)	1 (1.9%)	Unrelated
Investigations			
Elevated aminotransferase levels	2 (3.9%)	0	Possible
Elevated creatinine	1 (1.9%)	0	Possible
General disorders			
Fatigue	2 (3.9%)	2 (3.9%)	Possible
Headache	2 (3.9%)	0	Possible
Presyncope	1 (1.9%)	1 (1.9%)	Possible
Metabolism and nutrition disorders			
Hyperlipidemia	1 (1.9%)	0	Possible
Others			
Breast pain	1 (1.9%)	0	Possible
Brain aneurysm	1 (1.9%)	0	Unrelated

## Data Availability

Our dataset for the final manuscript and Supporting Information section has been successfully uploaded to NIH GEO; its accession number is GSE285765.

## Supporting Information

Supporting Information Additional supporting information can be found online in the Supporting Information section. Description of modeling and visualization of cell proliferation signaling after HT using the STRING database studying four hallmark gene sets related to cell proliferation: TNFA, TGF beta, PI3K/AKT/MTOR, and P53 pathways. Description of how RNA sequencing and NanoString nCounter data was analyzed. Changes in Wnt, Notch, oxidative stress–induced senescence, and cell proliferation pathways after HT. Table S1: List of 28 genes analyzed in multiplex analysis using NanoString nCounter analysis. Table S2: Mean percent decrease in max VBD% grouped on the basis of age and max VBD% at baseline. Table S3: Mean change in max VBD% grouped on the basis of menopausal status. Table S4: Number and percentage of patients in each category of age and max VBD% at baseline with decreased breast density after hydroxytyrosol. Figure S1: A box plot comparing percent decrease in breast density between baseline VBD groups for postmenopausal patients.
