# Supplementary material for: Hydroxytyrosol, a Component of Olive Oil for Breast Cancer Prevention in Women at High Risk of Cancer
Source: Int J Breast Cancer. 2025 Jan 21;2025:8831168. doi: 10.1155/ijbc/8831168 (PMC11774573; doi:10.1155/ijbc/8831168)

**SUPPLEMENTARY DOCUMENTS**

**Modeling and Visualization of Cell Proliferation Signaling Significantly Changed after HT**

We identified four hallmark gene sets related to cell proliferation from the fifty hallmark gene sets recorded in GSEA Human MSigDBv2022.1 database. The gene sets, namely HALLMARK_TNFA_SIGNALING_VIA_NFKB, HALLMARK_TGF_BETA_SIGNALING, HALLMARK_PI3K_AKT_MTOR_SIGNALING and HALLMARK_P53_PATHWAY, had 78 unique genes with differential expression p-value<=0.05 from DESeq (See Supplemental document for details).

These 78 genes were used to query STRING database and a single figure was created with each gene represented by a node, as described in the previous section. The face of each node was equally split into four sectors, with the sector in the first quadrant indicating the membership of TNFA hallmark gene set, the second quadrant indicating the membership of TGF_BETA gene set, the third quadrant indicating the membership of PI3K_AKT_MTOR gene set and the fourth quadrant for P53 gene set.

**Cell Proliferation Signaling after HT**

We identified four hallmark gene sets related to cell proliferation from the fifty hallmark gene sets recorded in GSEA Human MSigDBv2022.1 database.

The unique genes in total with differential expression p-value<=0.05 from DESeq are as follows:

- HALLMARK_TNFA_SIGNALING_VIA_NFKB, 167 of 200 member genes expressed in the dataset, 16 genes with p-value<=0.05, with 13/16 genes down-regulated EOT;
- HALLMARK_TGF_BETA_SIGNALING, 50 of 54 member genes expressed in the dataset, 11 genes with p-value<=0.05, with all 11 genes down-regulated EOT;
- HALLMARK_PI3K_AKT_MTOR_SIGNALING, 92 of 105 member genes expressed in the dataset, 28 genes with p-value<=0.05, with 27/28 genes down-regulated EOT;
- HALLMARK_P53_PATHWAY, 184 of 200 member genes expressed in the dataset, 27 genes with p-value<=0.05, with 24/27 genes down-regulated EOT;

Among the above mentioned significantly changed genes, TSC22D1 and LIF belonged to both TNFA and P53 gene sets, while MAP3K7 and UBE2D3 belonged to both TGF_BETA and PI3K_AKT_MTOR gene sets, while all other genes belonged to one gene set, making it 78 unique genes in total across four hallmark gene sets related to cell proliferation.

**RNA Sequencing & NanoString nCounter Analysis**

There were 44,579 unique transcripts identified in at least one of the 32 samples, and 15,768 transcripts had at least 15 reads in more than 75% of the samples. There were 3,330 transcripts with p-value <=0.05 which were differentially expressed, 190 of these were upregulated and 90 were downregulated in EOT vs. BL by at least 1.5-fold. With the cutoff set as “hypergeometric test p-value<=0.01” and “more than 2 member genes with significant changes”, there were 94 pathways overrepresented with EOT downregulated genes, associating with 34 of 90 downregulated genes.

There were two levels of statistical tests applied to the RNAseq data.

- In the gene level, Wald tests were applied via R package DESeq to identify differentially expressed genes in treated (EOT) vs. non-treated (BL) group, and only genes with BOTH p-value<=0.05 and fold change>=1.5 were selected. These genes consisted of less than 2% of genes passing the QC cutoffs (260 out of 15,768).
- In the pathway level, Hypergeometric tests with Benjamini-Hochberg adjustment for false discovery rates were calculated, plus at least any significantly overrepresented pathway needed to have at least 2 member genes in the list of significantly changed genes.

***Wnt Signaling Pathway Downregulated by Hydroxytyrosol***

Two of the top 25 pathways overrepresented with EOT downregulated are related to Wnt signaling, i.e., R-HSA-195721 (Signaling by Wnt p-value 0.0001587, q-value 0.001439) and R-HAS-201681 (TCF dependent signaling in response to Wnt, p-value 0.000151, q-value 0.001438). Further review of expression profiles for all 261 member genes in R-HSA-195721 confirmed that 82 of 261 member genes had p-value<0.05 for EOT vs. BL, among which only 4 were up in EOT and 78 were down in EOT condition.

The 82 genes were highly interactive with each other (Figure 4A), with 379 high confidence PPIs (confidence score>0.7 from STRING database) involving 81 of these 82 genes; only one gene, SOX13, did not have any PPIs within this group. There were two highly inter-connected clusters, one involving 16 genes mostly from Phenol-soluble modulin family, with at least 10 PPIs within the cluster; and the other with 22 genes features mostly histone genes, with at least 14 PPIs within the cluster. Genes shared with other two pathways, i.e., R-HSA-157118: Signaling by NOTCH and R-HAS-2559580: Oxidate Stress induced Senescence, were highlighted by red boundaries around the node. This overlapped subgroup includes all 16 genes from the Phenol-soluble modulin family, 16/22 genes related to histones, as well as CUL1, RBX1, SKP1 and YWHAZ.

***Notch Signaling Pathway Downregulated by Hydroxytyrosol***

R-HSA-157118: Signaling by NOTCH was significantly overrepresented with EOT downregulated genes (p-value 0.007983, q-value 0.02228), with 54 of 247 member genes were downregulated (p-value<0.05) for EOT vs. BL (Figure 4B). The 54 genes were highly interacting with each other, with 323 high confidence PPIs (confidence score>0.7 from STRING database) involving 50 of these 54 genes. 36 of these 54 member genes were shared with WNT pathway.

***Oxidative Stress Induced Senescence Pathway Downregulated by Hydroxytyrosol***

R-HSA-2559580: Oxidate Stress induced Senescence was significantly overrepresented with EOT downregulated genes (p-value 0.0001669, q-value 0.001439), with 32 of 126 member genes were downregulated (p-value<0.05) for EOT vs. BL (Figure 4C). The 32 genes were highly interacting with each other, with 149 high confidence PPIs (confidence score>0.7 from STRING database) involving 30 of these 32 genes. 17 of these 32 member genes were shared with Wnt pathway.

***Cell Proliferation Signaling Significantly Downregulated after Hydroxytyrosol***

The four hallmark gene sets discussed in “Methods section” were analyzed using DESeq and 78 unique genes with differential expression p-value<=0.05 (Figure 5), with 72/78 genes downregulated at EOT. There were 110 high confidence PPIs (confidence score>0.7 from STRING database) involving 51 of these 78 genes. Therefore, Hydroxytyrosol may impact cell proliferation by interacting with subnetworks across multiple pathways by downregulating the overall cell proliferation process.

**Visualization of Signaling based on Significantly Downregulated Pathways after HT**

Given the 94 pathways overrepresented with EOT downregulated genes, we identified three pathways from REACTOME database to model the signaling subnetworks responding to HT. For the three pathways, R-HSA-195721: Signaling by WNT, R-HSA-157118: Signaling by NOTCH, R-HAS-2559580: Oxidate Stress induced Senescence, we obtained the lists of member genes from GSEA Human MSigDB v2022.1 database. For each pathway, the member genes with differential expression p-value<=0.05 from DESeq used to query STRING database version 11.5. High confidence PPIs with combined confidence score>=0.7 were collected and the resulting PPI networks were visualized using Cytoscape 3.9.1. Three figures for the three pathways were created where each member gene was represented by a circle node. The size of a node was proportional to the number of PPIs, and the node face color was proportional to a significant score defined as $-{log}_{10}\left( p\left( EOT vs. BL \right) \right)*Sign$, where $p\left( EOT vs. BL \right)$ is the differential expression p-value for EOT vs. BL generated by DESeq, and Sign=-1 if EOT<BL and 1 otherwise. The member genes shared between Wnt and Notch pathways were highlighted by red boundaries around the nodes in these two figures; all nodes with same boundaries in the figure for Oxidate Stress induced Senescence were shared among all three pathways.

**Table S1:** List of 28 genes analyzed in multiplex analysis using NanoString nCounter analysis

| AKT1 | FRAT1 | MAPK1 | TCF7 | WNT10B | WNT2B | WNT5B |
| --- | --- | --- | --- | --- | --- | --- |
| CCND1 | FZD1 | PIK3CA | WIF1 | WNT11 | WNT3 | WNT6 |
| DVL1 | LRP5 | PORCN | WNT1 | WNT16 | WNT4 | WNT7A |
| DVL2 | LRP6 | PPARD | WNT10A | WNT2 | WNT5A | WNT7B |

**Table S2:** The mean percent decrease in Max VBD%

| **Age** | **Max VBD% at Baseline** | | |
| --- | --- | --- | --- |
|  | **< 10% (N=14)** | **≥ 10% (N=12)** | **Total (N=26)** |
| **< 60 (N=15)** | -18.79% | -5.67% | -10.04% |
| **≥ 60 (N=11)** | 2.44% | 5.23% | 3.71% |
| **Total (N=26)** | -7.21% | -2.04% | -4.22% |

**Table S3:** The mean percent decrease in breast density as measured by change in max VBD%

| **Menopausal Status** | **VBD Max % at Baseline** | | |
| --- | --- | --- | --- |
|  | **< 10% (N=14)** | **≥ 10% (N=12)** | **Total (N=26)** |
| **Pre (N=10)** | -8.65% (N=2) | -8.93% (N=8) | -8.90% |
| **Post (N=16)** | -7.07% (N=12) | 5.84% (N=4) | -1.75% |
| **Total (N=26)** | -7.21% | -2.04% | -4.22% |

**Table S4:** The percentage of subjects in each of the categories experiencing a decrease in breast density.

| **Age** | **Max VBD% at Baseline** | | |
| --- | --- | --- | --- |
|  | **< 10% (N=14)** | **≥ 10% (N=12)** | **Total (N=26)** |
| **< 60 (N=15)** | 20.0% | 50.0% | 40.0% |
| **≥ 60 (N=11)** | 66.7% | 80.0% | 72.7% |
| **Total (N=26)** | 45.5% | 60.0% | 53.8% |

**Figure S1**: Box Plot comparing Percent Decrease in Breast Density between Baseline VBD Groups for Post-Menopausal Subjects.


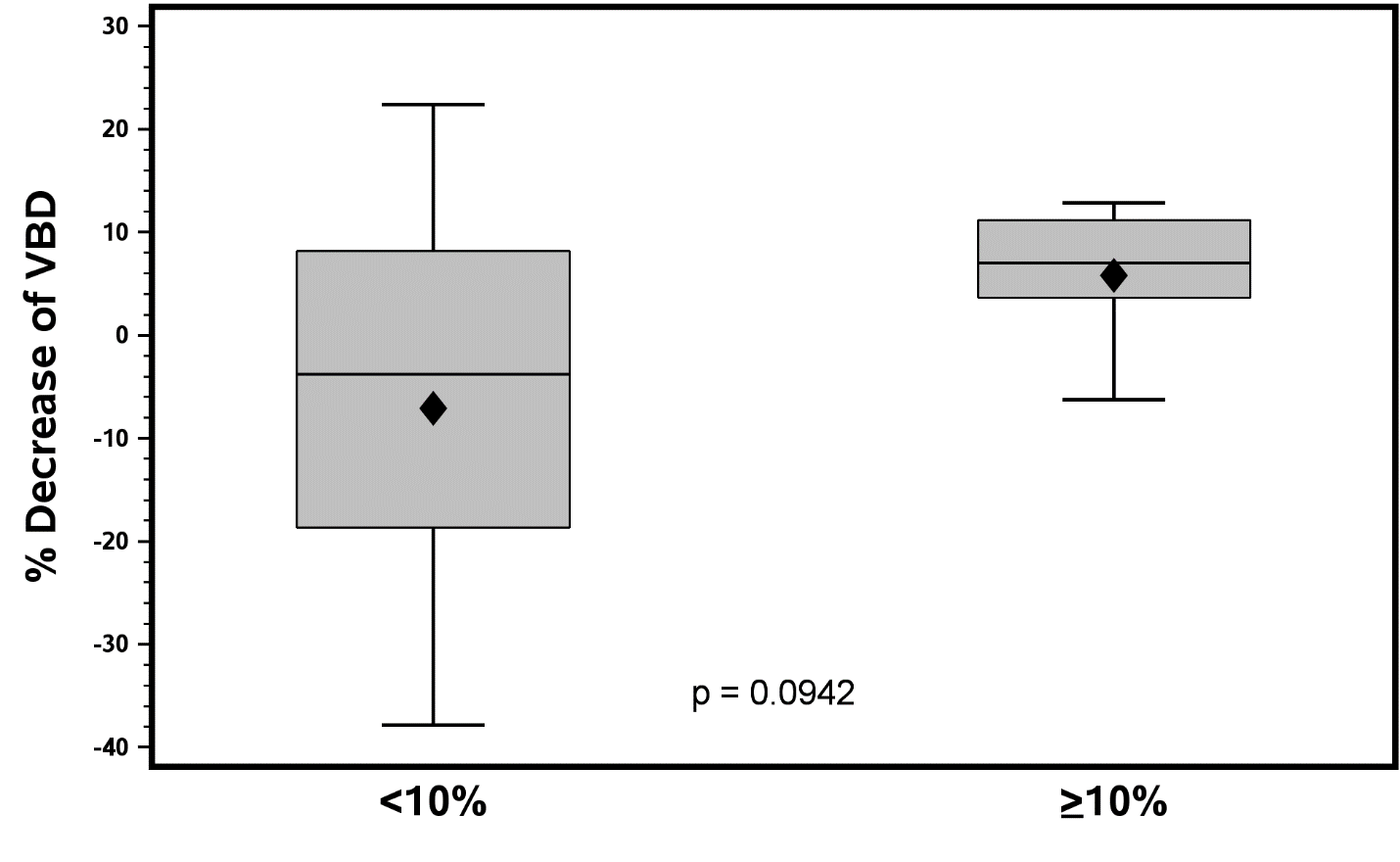

Supplement: Supporting Information — Additional supporting information can be found online in the Supporting Information section. Description of modeling and visualization of cell proliferation signaling after HT using the STRING database studying four hallmark gene sets related to cell proliferation: TNFA, TGF beta, PI3K/AKT/MTOR, and P53 pathways. Description of how RNA sequencing and NanoString nCounter data was analyzed. Changes in Wnt, Notch, oxidative stress–induced senescence, and cell proliferation pathways after HT. Table S1: List of 28 genes analyzed in multiplex analysis using NanoString nCounter analysis. Table S2: Mean percent decrease in max VBD% grouped on the basis of age and max VBD% at baseline. Table S3: Mean change in max VBD% grouped on the basis of menopausal status. Table S4: Number and percentage of patients in each category of age and max VBD% at baseline with decreased breast density after hydroxytyrosol. Figure S1: A box plot comparing percent decrease in breast density between baseline VBD groups for postmenopausal patients. [file 8831168.f1.docx]
